# Interference in Ballistic Motor Learning: Specificity and Role of Sensory Error Signals

**DOI:** 10.1371/journal.pone.0017451

**Published:** 2011-03-09

**Authors:** Jesper Lundbye-Jensen, Tue Hvass Petersen, John C. Rothwell, Jens Bo Nielsen

**Affiliations:** 1 Department of Neuroscience and Pharmacology, University of Copenhagen, Copenhagen, Denmark; 2 Department of Exercise and Sport Sciences, University of Copenhagen, Copenhagen, Denmark; 3 Sobell Department of Motor Neuroscience and Movement Disorders, Institute of Neurology, University College London, London, United Kingdom; Harvard Medical School, United States of America

## Abstract

Humans are capable of learning numerous motor skills, but newly acquired skills may be abolished by subsequent learning. Here we ask what factors determine whether interference occurs in motor learning. We speculated that interference requires competing processes of synaptic plasticity in overlapping circuits and predicted specificity. To test this, subjects learned a ballistic motor task. Interference was observed following subsequent learning of an accuracy-tracking task, but only if the competing task involved the same muscles and movement direction. Interference was not observed from a non-learning task suggesting that interference requires competing learning. Subsequent learning of the competing task 4 h after initial learning did not cause interference suggesting disruption of early motor memory consolidation as one possible mechanism underlying interference. Repeated transcranial magnetic stimulation (rTMS) of corticospinal motor output at intensities below movement threshold did not cause interference, whereas suprathreshold rTMS evoking motor responses and (re)afferent activation did. Finally, the experiments revealed that suprathreshold repetitive electrical stimulation of the agonist (but not antagonist) peripheral nerve caused interference. The present study is, to our knowledge, the first to demonstrate that peripheral nerve stimulation may cause interference. The finding underscores the importance of sensory feedback as error signals in motor learning. We conclude that interference requires competing plasticity in overlapping circuits. Interference is remarkably specific for circuits involved in a specific movement and it may relate to sensory error signals.

## Introduction

It is evident that the central nervous system has an impressive capability of forming and maintaining multiple long-term motor memories. We can acquire different motor skills such as bicycling, ice skating or driving a car and once acquired, these skills are often retained for a very long time [1]. However, despite the versatility of our motor repertoire learning may be hindered and interference can occur if we engage in subsequent learning of different motor skills [2], [3].

Practice of a new motor task, A, leads to improved performance which can last many hours and days after practice. Immediately after practice, the “motor memory” of A is fragile and retrograde interference may occur if another task, B, is learned shortly afterwards [2], [4]. In some instances, the memory of task A becomes more resistant to interference over time, such that practice of task B on the following day no longer disrupts the memory of A [5], [6]. Consolidation is defined as the process, by which motor memories become increasingly stable with continued passage of time, and one mechanism of interference is disruption of consolidation [5]. In other cases, where it has been postulated that task B is viewed as a variant of task A, such as force-field adaptation or learning of one form of visuomotor rotation followed by another rotation through a different angle, then interference occurs between A and B even on day 2 despite the fact that A had been well learned on day 1 [6], [7], [8], [9]. This persistent interference has practical value. When we have two successive lessons practising the same skill (e.g. a golf swing), it makes some sense that the system should view them as a single continuous process of skill acquisition rather than two separate memories. It may be that in this case, performance of task B on day 2 re-engages the motor memory of task A, which then becomes susceptible to interference. Contextual cues (the feel of the golf club) have been speculated as one mechanism that might allow the brain to distinguish between different internal models and thereby learning the same versus different tasks [9], [10].

In many studies on motor interference there has been an assumption that early interference occurs because the motor output required for the second task interacts with the motor representation of the previously learned task i.e. retrograde interference. This is consistent with the fact that interference after learning task A only occurs if task B is novel and being learned; it does not occur if we perform our normal daily activities. Presumably we cannot interfere with a memory unless we actively engage the system of memory formation itself. The mechanisms of interference may relate to disruption of motor memory consolidation (retrograde interference) or to persisting representations of previously learned motor skills (anterograde interference) [11]. A recent view is that switching between multiple motor skills may be problematic due to contextual retrieval effects [6], [10]. However, little is known about what details of the new task are actually necessary for interference to occur. Here we ask what factors determine whether or not tasks will interfere with each other. In particular: (1) do the motor memories of A and B have to involve the same movement direction and muscles or just the same joints? (2) is the interference purely motor, or does the sensory input that is being used to improve performance on task B also play a role?

The present experiments concern the case in which learning two different skills at the same joint shows early but not late interference. We propose that during repeated practice of a task, there are relatively rapid changes in the effectiveness of synapses in neural circuits that control the movement. Stabilisation of these changes to produce long-term modifications in transmission requires protein synthesis that may take several hours [2], [12]. During this period, if the cellular network is reengaged in another session of learning then consolidation of the first task will be disrupted. If this is true we predict that interference should be movement specific and occur only if the neurones that are engaged in the learning of that task are also engaged in learning the second task.

**Figure 1:**
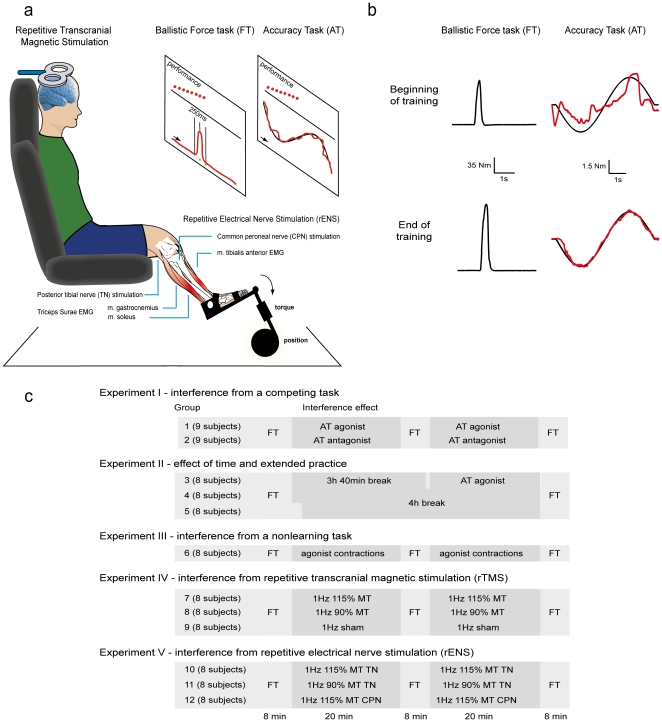
Methodological overview and experimental conditions. (**a**) The primary learning task was a ballistic force task (FT) performed as isometric ankle plantar flexion. In order to study interference effects practice of the primary task was interleaved with periods involving different activities e.g. practicing an accuracy-tracking task (AT). (**b**) Depicts the motor performance of a subject from Group 2 at the beginning and at the end of ballistic force task and accuracy task practice respectively. For the force task, the black traces represent plantar flexion torque. For the accuracy task black represents the target while red represents the exerted torque. (**c**) Subjects were divided in 12 groups. Practice consisted of FT learning periods of 8 minutes separated by a period of 20 min. or ∼4 hours. During the breaks subjects either practiced a secondary accuracy task (AT) with the FT agonist or antagonist muscles, performed a non-learning task, waited, received 1 Hz repetitive transcranial magnetic stimulation (rTMS) of the primary motor cortex or 1 Hz repetitive electrical nerve stimulation (rENS) of the agonist tibial nerve (TN) or the antagonist common peroneal nerve (CPN).

We developed an accuracy-tracking task (AT) (fig. 1A) at the ankle that could be performed using the plantar flexor muscles triceps surae (soleus (SOL) and gastrocnemius), or the dorsiflexor muscle, tibialis anterior (TA). We then tested what signals might be important in interference by examining separately the contribution of activity in motor output pathways to, and sensory inputs from the plantar flexor muscles. As predicted, movement-specific interference was observed with a previously acquired ballistic force task (FT) involving the plantar flexor muscles (fig. 1A): Interference was observed when the accuracy-tracking task was performed with the same agonist muscles and the same movement direction as the ballistic task. In contrast, interference was not observed when the identical competing task was performed as the opposite movement direction involving the antagonist muscles. Furthermore, interference required learning to occur and sensory input from the trained muscles or movement was surprisingly effective in causing interference, whereas motor output in the absence of sensory input (subthreshold repetitive transcranial magnetic stimulation (rTMS) to the primary motor cortex (M1)) was ineffective. The present study is, to our knowledge, the first to demonstrate that peripheral nerve stimulation may cause interference. We argue that this is because sensory feedback constitutes an important error signal in motor learning. Interference was not observed when the competing task was practiced 4 hours after initial motor practice suggesting that one possible mechanism in the observed interference effects may be disruption of consolidation.

## Results

A protocol allowing a direct test of both motor learning and interference effects was established. Participants were divided into 12 groups (fig. 1C) who all practised making ballistic pulses of maximal voluntary plantar flexion torque at the ankle in 2 or 3 sets of 35 trials (FT1, FT2 and FT3) separated by breaks of 20 minutes or 4 hours (fig. 1A). In the first set of trials, all groups improved their ability to generate maximal ballistic plantarflexor torque (fig. 1B) (mean ± s.e.m. increase from first to last trial) 32±3% (F_1,194_ = 122.7, p<0.001). There were no differences between groups (F_11,194_ = 0.129, p = 0.998). During the breaks different groups were exposed to different interventions (fig. 1C). Subjects were also able to improve motor performance in the subsequent practice sessions, but the retention of the behavioural improvement markedly depended on the interventions. The subjects in Group 1,2 and 6–12 participated in 2 experimental sessions with a minimum of 2 weeks in-between (see Methods section). When analyzing the baseline performance in theses experiments we observed that in session 2, baseline was 18±7% higher than in session 1 (t = 9.68 p<0.01). This demonstrates long-term retention of the ballistic force task learning.

### Experiment I: Interference with retention of motor learning is specific for direction of movement

Experiment I investigated the specificity of between-task interference. In the breaks between ballistic training (FT) sets, two groups of subjects practiced an accuracy task (AT) that involved tracking a moving target on a computer screen by generating low force. The task was identical for the two groups, but in Group 1, tracking was achieved by activating the plantar flexion muscles, whereas in Group 2, the tracking force was achieved by the opposite movement direction activating the dorsiflexor muscles (fig. 1A and 1C).

**Figure 2:**
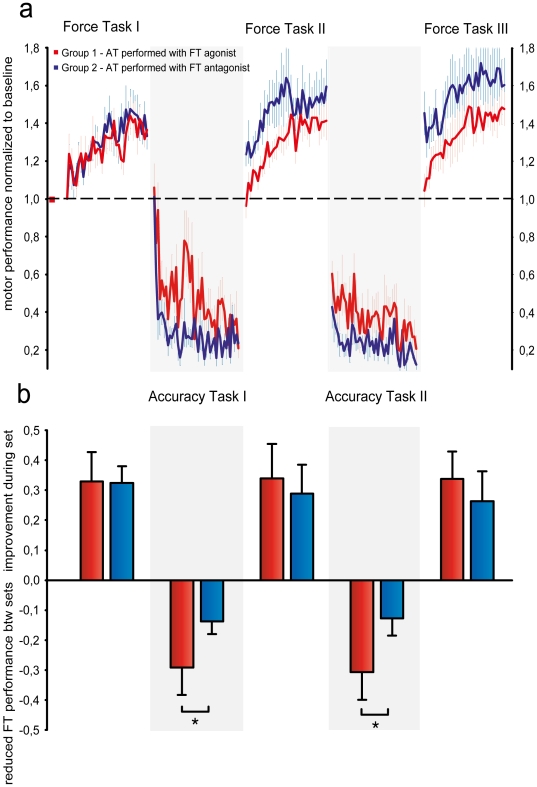
Between-task interference is movement and effector specific. Learning of the ballistic force task (FT), the accuracy task (AT) and between-task interference effects. (**a**) Learning curves for the two tasks. The ballistic force task was performed as plantar flexion whereas the accuracy task was performed as either plantar flexion (Group 1 – red) or dorsiflexion (Group 2 - blue). Motor performance was normalized to baseline (initial) ballistic force and deviation (error) from optimal tracking target respectively. During practice subjects increased ballistic force in FT and decreased deviation in AT. Curves represent group average motor performance, error bars represent s.e.m (**b**) Increase in FT motor performance during FT practice and decrease in FT performance during AT practice. Bars represent group average ± s.e.m. An asterisk denotes significant difference (p<0.05) in Bonferroni corrected tests.

The results obtained in Experiment I are illustrated in Figure 2. In order to test for differences in FT performance within and between intervention groups and training sets, data for individual subjects were entered into a two-way analysis of variance (ANOVA) with GROUP (Group1, 2, 6 and 9) and SET (change in motor performance during FT1, FT2, FT3 and between FT1-FT2 and FT2-FT3) as factors. The ANOVA yielded a significant main effect for SET F_(4, 169)_ = 38.95, p<0.001. The main effect of group was non-significant, F_(3, 169)_ = 1.245, p = 0.28. However, the GROUP×SET interaction was significant, F_(12, 150)_ = 2.743, p = 0.006, signifying that the changes in ballistic motor performance within and between sets was different between intervention groups.

Post hoc pairwise comparisons revealed that there were no between-group differences in FT acquisition within FT1 but between FT1 and FT2 ballistic motor performance decreased 29±9% in Group 1, meaning that in the second set of ballistic trials their first trial was 98% of baseline performance. This decrease in FT performance after AT practice was significantly different from Group 2 (t = 2.417, p = 0.048) in which ballistic performance only decreased 13,8±4%. Although both Group 1 and 2 had to track an identical object in the accuracy task, only Group 1 in which both FT and AT was performed with the plantar flexor muscles showed catastrophic interference with the force task (Fig. 2). During FT2, both Group 1 and 2 again improved motor performance by 34±11% and 29±10% respectively with no difference between groups (t = 0.107, p = 0.9), but the same pattern of interference was however observed following AT2. Group1, which practiced AT using plantarflexion, produced 31±10% less force in the first trial of FT3 than on the last trial of FT2 set 2. This effect was significantly different from Group 2 (t = 2.843, p = 0.014), in which FT only decreased 12.7±6%. During FT3 both groups again improved motor performance 34±9% and 26±9% of baseline with no difference between groups (t = 1.252, p = 0.7).

These results indicate that practice of the accuracy task lead to interference selectively in Group 1 when the two competing tasks engaged the same movement direction and the same agonist muscles. However, since both Group 1 and 2 were exposed to potentially interfering interventions inbetween FT sets, comparison of differences between these groups only allows interpretations on relative interference. Consequently, the ANOVA also included data from Group 9, which served as a control group in which subjects were only exposed to a sham intervention. Pairwise comparison of the effects observed in Group 1 and 2 to Group 9 revealed significant differences. Between FT1 and FTII the decrease in Group 9 motor performance was significantly different from Group 1 (t = 3.287, p = 0.003) but not Group 2 (t = 0.94, p = 0.93). Between FT2 and FT3 motor performance also decreased significantly more in Group 1 than Group 9 (t = 2.558, p = 0.047) whereas there was no difference between Group 2 and 9 (t = 0.4, p = 0.82). These results confirm that interference was selective for Group 1 in which the competing tasks involved the same movement direction and agonist muscles.

Analysis of the parameter estimates obtained for the individual FT learning curves (y = y0×ax^b^) demonstrated significant effects of both SET (F_2, 107)_ = 43.25, p<0.001) and GROUP (F_(3,107)_ = 4.26, p<0.01) on *y0* and also a significant GROUP×SET interaction (F_(6,96_ = 3.42 p<0.01). In Group 1 there was no difference in *y0* between sets indicating that there was no retention and interference was complete. In contrast, *y0* increased significantly from FT1 to FT2 (t = 2.198, p = 0.055) and FT 2 to FT3 (t = 4.12, p = 0.014) in Group 2 signifying significant FT retention after AT practice. This was also the case for Group 9 from FT1 to FT2 (t = 2.817, p = 0.01) and FT2 to FT3 (t = 2.43, p = 0.023). Within FT2 there was a significant difference in *y0* between Group 1 and 2 (t = 2.64, p = 0.017) and between Group 1 and 9 (t = 2.76, p = 0.016). This was also the case for FT3 in which *y0* was higher for both Group 2 (t = 3.033, p<0.019) and Group 9 (t = 4.27, p<0.01) compared to Group 1. Although the parameter estimate *a* decreased slightly between sets in Group 2 and 9 whereas it remained constant in Group 1 no significant differences were observed.

The results demonstrate a remarkable specificity of interference in motor learning. In Group 1, complete interference and no FT retention was observed after competing AT training involving the same agonist muscles and movement direction. In contrast, practice of the competing task involving the antagonist muscles and opposite movement direction did not cause interference.

There were also significant differences between groups in retention of the accuracy task. The two-way ANOVA performed on changes in AT performance within and between sets yielded a significant main effect for SET F_(3, 71)_ = 56.52, p<0.001, and the GROUP×SET interaction was also significant, F_(3, 64)_ = 10.06, p<0.001. Following FT1, the initial error in AT1 increased slightly in Group 1 compared to baseline. This effect was however not significantly different between groups (t = 2.433, p = 0.19). Within AT1, both groups reduced tracking error with no difference between groups (t = 2.955, p = 0.122), but between AT1 and AT2 tracking error increased significantly more in Group 1 than Group 2 (t = 3.343, p = 0.039). In AT2 both groups again improved accuracy.

Thus, for both tasks, interference was strong and specific in the group which used plantar flexion in the tracking task as well as the ballistic task, whereas there was no interference if the tracking task was performed as dorsiflexion involving the antagonist muscles and movement direction.

### Experiment II: Ballistic motor learning consolidates over time and with increased initial training; Passage of time hinders between-task interference

**Figure 3:**
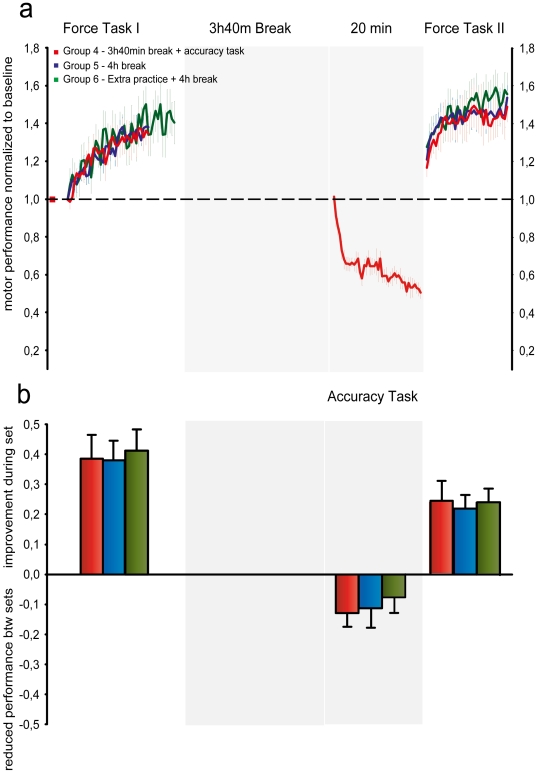
Ballistic motor learning consolidates over time and with increased initial training; Passage of time hinders between-task interference. (**a**) Learning curves for the FT task. Group 3 (red) practiced the competing accuracy task after a 3 h40 min break. Group 4 (blue) had 4 h break. Group 5 (green) had extra initial practice and 4 h break. Performance was normalized to baseline. Curves represent group average FT motor performance. Error bars represent s.e.m. (**b**) Increase in FT motor performance during practice and decrease in FT performance during breaks. Bars represent group average ± s.e.m.

Previous studies have suggested that interference between motor tasks may relate to disruption of early consolidation following motor learning [2], and this has also been found for a ballistic task as in the present study [5], [13]. It is however also possible that interference occurs through other (largely unknown) mechanisms and recent studies of sensorimotor adaptation have failed to demonstrate that time for consolidation stabilizes motor memories against interference [7], [8]. In Experiment II we allowed 4 hours to pass in between FT training sets. Group 3 had 3 hours and 40 minutes break after initial FT training. Following this break subjects engaged in AT training involving the same agonist muscles and movement direction (i.e. plantar flexion) corresponding to Group 1. Immediately after this the subjects engaged in FT2. Subjects in Group 4 did not practice the competing AT task. Instead these subjects had a break of 4 hours between FT sets. In Group 5 subjects also had 4 hours break between FT sets but the amount of training in FT1 was increased from 35 trials to 45 trials in order to investigate whether the learning consolidated with an increased amount of initial training (Fig. 3).

For FT performance, a 2-way ANOVA was used to test for changes within and between groups during FTI, FTII and between FT sets. This test revealed a significant main effect of set (F_2,71_ = 103.31, p<0.001), no significant main effect for group (F_2,71_ = 0.0125, p = 0.99) and a tendency to a GROUP×SET interaction (F_4,63_ = 2.13, p = 0.08). During initial ballistic practice (FT1), Group 3–5 improved performance with no significant differences between groups. Group 3 improved FT performance by 37±7%, Group 4 improved performance by 36±6% and Group 5 improved performance by 41±7%. Analysis of the individual learning curves also revealed no differences between groups. 3 hours and 40 minutes after initial FT practice Group 3 engaged in AT practice leading to significantly reduced tracking errors (t = 20.81, p<0.001). Figure 3b shows that after a break of 4 hours between FT training sets there was retention of the learning in Group 4. Contrary to what was observed in Experiment I, where learning of a competing task immediately after initial learning caused interference, no interference was observed for Group 3, in which subjects engaged in AT learning 3 hours and 40 minutes after initial FT practice. There was no significant difference between Group 3 and 4 in the change in FT motor performance between FT1 and FT2 (t = 0.11, p = 0.98. Analysis of the learning curve parameter estimates demonstrated a significant main effect of SET (F_1, 47)_ = 37.44, p<0.001) on *y0* and a GROUP×SET interaction (F_(2,42_ = 1.136, p = 0.331). In both Group 3 (t = 3.049, p = 0.02), 4 (t = 3.265, p = 0.018) and 5 (t = 3.993, p<0.01) *y0* increased significantly from FT1 to FT2. The parameter estimate *a* decreased between FT1 and FT2 (main effect F_1,47_ = 4,37, p<0.01) with no significant differences between groups. These findings indicate that a break of almost 4 hours between learning sessions prevents the learning of a competing AT task from causing interference.

In Group 5 we found a tendency that extended practice in the initial training set was followed by a smaller drop in performance during the break compared to Group 3 (t = 1.97, p = 0.068 and group 4 (t = 1.86, p = 0.08). When additionally comparing FT performance at the beginning and end of FT1 and FT2 for Group 3–5 in an additional two-way ANOVA we found a significant SET×GROUP interaction (F_6,84_ = 3.298, p = 0.03) revealing that at the beginning of FT2 Group 5 had a significantly higher FT motor performance than Group 3 (t = 2.651, p = 0.017) and 4 (t = 2.453, p = 0.025). This finding seems consistent with the notion that saturation learning improves retention [9]. During the second ballistic force task training set all 3 groups improved performance with no significant differences between groups.

### Experiment III: Interference with retention of motor learning is not seen with a simple nonlearning task

**Figure 4:**
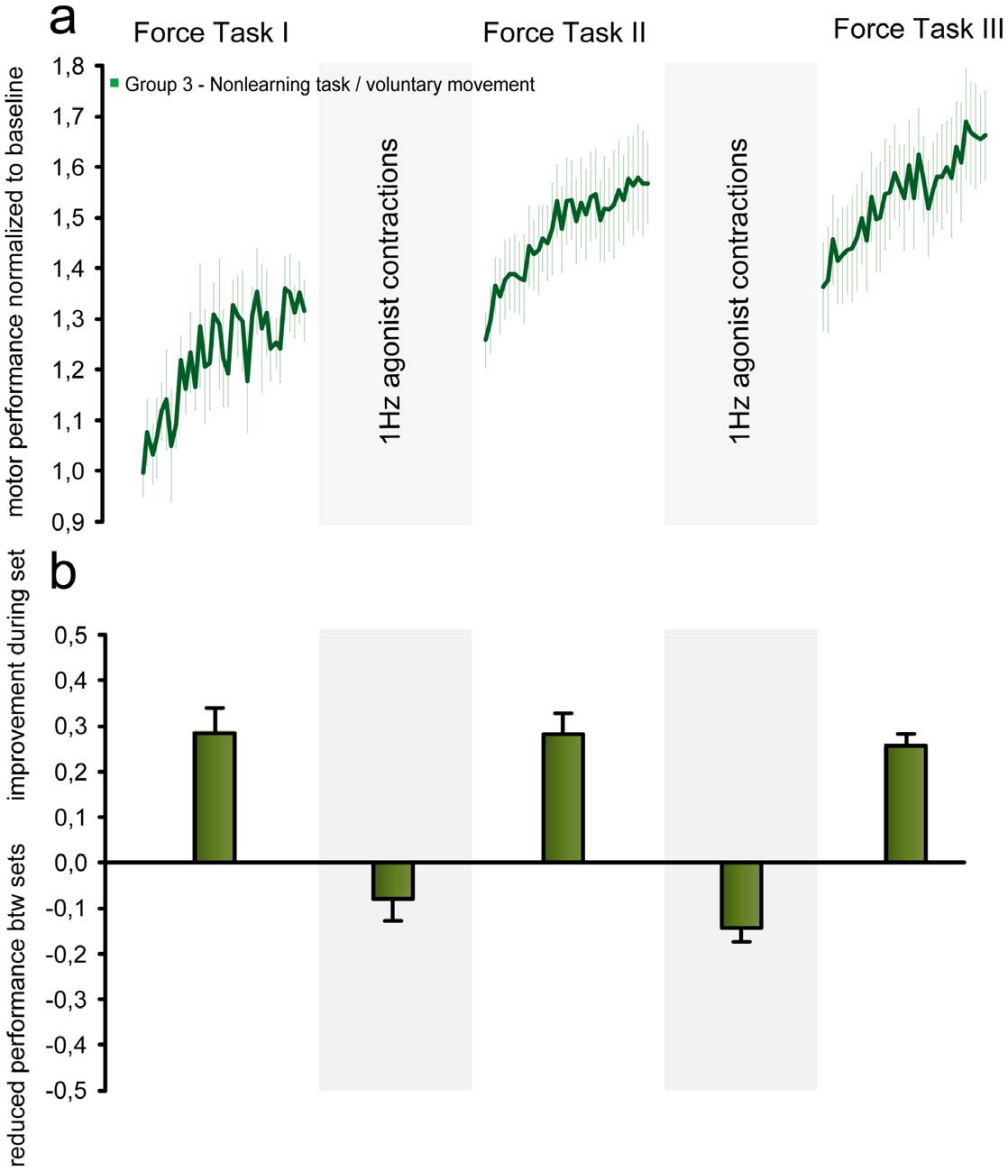
Performing a non-learning task does not lead to interference. **a**) Learning curves for the FT task. Performance was normalized to baseline. Curves represent group average FT motor performance Error bars represent s.e.m During breaks subjects performed a non-learning task involving voluntary 1 Hz agonist contractions. (**b**) Increase in FT motor performance during practice and decrease in FT performance during breaks. Bars represent group average ± s.e.m.

In the interval between ballistic training sets, Group 6 (Fig. 4) performed plantar flexions at 1 Hz by approximately the same amount as in the ankle-tracking task, but without being required to be as precise as possible and without any visual feedback on motor performance (Figure 4A). Changes in FT performance within and between sets were entered into the two-way ANOVA previously described for Experiment I in order to allow comparison between groups.

The decrease in motor performance observed between FT1 and FT2 (Fig. 4B) was significantly smaller in Group 6 compared to Group 1 (t = 3.291, p = 0.003) with no differences between Group 6 and Group 2 (t = 0.921, p = 0.83) or between Group 6 and Group 9 (t = 0.42, p = 0.89). The same tendency was observed between FT2 and FT3. Again, Group 6 FT performance decreased significantly less than Group 1 (t = 2.458, p = 0.047) with no difference to Group 2 and 9 indicating no interference. Analysis of the learning curves revealed an increase in baseline (*y0*) from the FT1 to FT2 (t = 3.071, p = 0.025) and from FT2 to FT3 (t = 2.751, p = 0.047). The slope parameter *a* decreased insignificantly between training sets.

Conclusively, performance of this non-learning task in the breaks between FT set failed to produce interference with retention of the ballistic task (Fig. 4). We conclude that the interference observed in Experiment I is not caused by extensive use of the agonist muscle i.e. fatigue. More likely, engagement in a task, which produces motor learning, is essential for interference to occur.

### Experiment IV: Suprathreshold rTMS causes interference – subthreshold rTMS does not

We were surprised that voluntary contractions without any requirement to acquire skill did not cause interference. Muellbacher et al. [5] and Baraduc et al. [13] previously examined a similar ballistic learning task in the hand and found that rTMS of the primary motor cortex (M1) at a similar rate (i.e. 1 Hz) and producing a similar amount of contraction as in our volitional task abolished retention. In Experiment IV we repeated their experiment by applying rTMS in the breaks between FT sets. In 2 groups of subjects 1 Hz rTMS was applied to M1 over the hotspot for ankle muscle activation at 115% (Group 7) and 90% (Group 8) of resting motor threshold (rMT). In Group 9 sham rTMS was applied.

**Figure 5:**
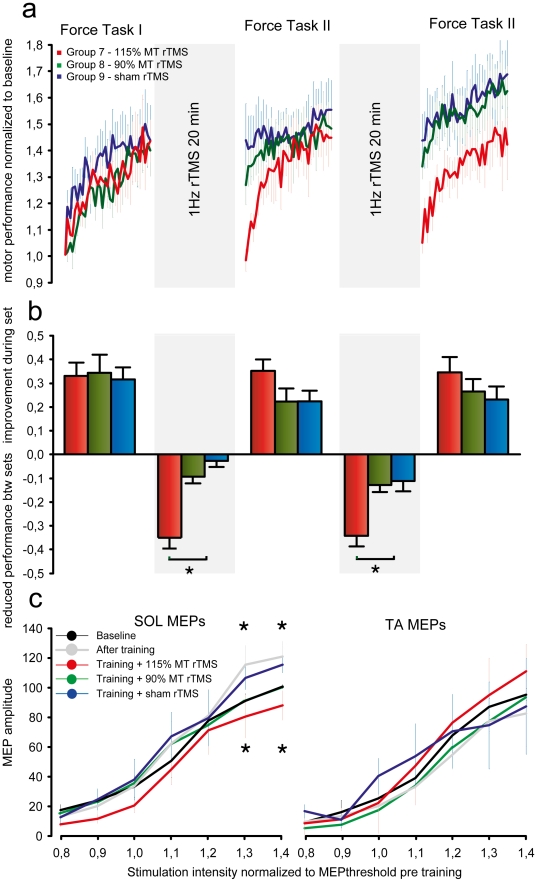
Suprathreshold but not subthreshold rTMS leads to interference. Effects of FT motor practice and rTMS during breaks on motor learning and corticospinal excitability. (**a**) Learning curves for the FT task. Performance was normalized to baseline. Curves represent group average FT motor performance Error bars represent s.e.m. During the breaks Group 7 (red) received 115% rMT rTMS of M1, Group 8 (green) received 90% rMT rTMS of M1 and Group 9 (blue) received 1 Hz sham rTMS. (**b**) Increase in FT motor performance during practice and decrease in FT performance during breaks. Bars represent group average ± s.e.m (**c**) Motor evoked potential (MEP) recruitment curves for agonist (SOL) and antagonist (TA) before (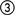) and after (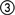) FT training, and after training + rTMS (red, green, blue). Abscissa represents stimulation intensity, the ordinate represents MEP amplitude normalized to MEP_max_ before training. An asterisk denotes significant difference (p<0.05) in Bonferroni corrected tests.

Figure 5 shows that rTMS at 115% rMT interfered with retention of the ballistic learning, whereas subthreshold rTMS and sham rTMS did not. Differences in individual FT motor performance within and between sets were entered into a two-way ANOVA for Group 7–9. This analysis revealed a main effect of SET (F_2,71_ = 135.87, p<0.001), a tendency to a main effect of GROUP (F_2,71_ = 2.575, p = 0.078) and a significant GROUP×SET interaction (F_4,63_ = 10.77, p<0.001). There were no differences between groups in FT1. During the first period of rTMS at 115% rMT, ballistic force decreased 34±5% to 98% of baseline in Group 7 indicating complete interference. This decrease in FT performance was significantly less in Group 8 (90% rMT TMS) (t = 4.963, p = 0.002) and Group 9 (sham rTMS) (t = 6.025, p<0.001) in which FT performance decreased 8±2% and 3±3%. No difference was observed between Group 8 and 9 (t = 1.061, p = 0.868) indicating that rTMS at 90% rMT did not cause interference.

After the second period of rTMS at 115% rMT, FT motor performance again decreased by 33±5% to 103% of baseline in Group 7. This was significantly different from Group 8 (t = 4.227, p<0.001) and Group 9 (t = 4.288, p<0.001) in which FT performance decreased 12±2% and 10±3% respectively with no significant difference between these groups (t = 0.256, p = 0.99).

Although rTMS at 115% rMT caused interference, the ability to improve FT performance with practice was not impaired. Both during FT2 and FT3 motor performance increased 35±5% and 35±6% with no difference to FT1 (t = 1.076, p = 0.98 and t = 0.263, p = 0.99). In Group 8 and 9 FT performance increased significantly less during FT2 compared to Group 7 (23±5%, t = 3.983, p<0.001 and 23±4% t = 3.698, p<0.001). The same tendency was found for FT3 during which FT performance increased 27±6% (t = 1.686, p = 0.195) and 23±6% (t = 2.12, p = 0.104) respectively.

Analysis of the individual learning curves revealed no differences between groups in the first ballistic training set. For *y0*, there was a significant main effect of both GROUP (F_2,71_ = 4.04, p<0.01) and SET (F_2,,71_ = 40.39, P<0.001) and a GROUP×SET interaction (F_4,,63_ = 3.51, p<0.01). With subsequent practice *y0* increased from FT1 to FT2 (t = 2.67, p = 0.016 and t = 3.14, p = 0.001) and from FT1 to FT3 (t = 2.75, p = 0.005 and t = 3.18, p<0.001) in the 90% rMT and sham rTMS groups. There were no differences in *y0* between sets in the 115% rMT group indicating no retention, but significant differences between the 115% rMT rTMS group and the other two groups in FT2 (t = 2.80, p = 0.004 and t = 3.12, p<0.001)) and FT3 (t = 2.84 p<0.001 and t = 3.03, p<0.001). The parameter estimate *a* displayed a significant main effect of SET (F_2,71_ = 3,37, p<0.01). For Group 8 and 9 the estimate of *a* tended to decrease in FT2 and FT3, but there were no significant differences between groups. In conclusion 1 Hz rTMS at 115% rMT caused complete interference and abolished retention of the ballistic learning whereas rTMS at 90% rMT did not cause interference.

We also examined corticospinal excitability before and after ballistic training by plotting the input-output relationships of motor evoked potential amplitudes (MEPs) in SOL and TA muscles. For SOL there was a main effect of STIMULATION INTENSITY (F_6,279_ = 38.75, p<0.001), GROUP (F_4,279_ = 6.11, p<0.01) and a significant STIMULATION INTENSITY×GROUP interaction (F_24,245_ = 2.60, p = 0.02). For TA there was only a main effect of STIMULATION INTENSITY (F_6,279_ = 23.42, p<0.001). Corticospinal excitability increased in SOL after ballistic training: MEPs were significantly facilitated at stimulation intensities of 1.3 (t = 2.84, p = 0.015) and 1.4 rMT (t = 2.47, p = 0.032). However, this increase was abolished by rTMS at 115% rMT as reported previously [5], [14], [15], [16] - for review see [17]: in fact SOL MEP amplitudes obtained at 1.3 rMT (t = 3.23, p<0.001) and 1.4 rMT (t = 3.15, p = 0.001) were significantly smaller than after FT practice alone. Although less pronounced, subthreshold rTMS at 90% MT was also accompanied by a decrease in MEP amplitudes compared to post training values (1.3 rMT t = 2.43, p = 0.042, 1.4 rMT t = 2.42 p = 0.048). Following sham rTMS there were no significant differences from post training values. Both the training induced facilitation and the depression of corticospinal excitability observed following rTMS was effector-specific since it was observed only for the agonist SOL but not for the antagonist TA (Fig. 5C).

### Experiment V: Repetitive electrical stimulation of the nerve to the trained muscle, but not its antagonist causes interference

Why did suprathreshold rTMS lead to interference whereas subthreshold rTMS and voluntary movement did not? In Experiment V we examined the effect of producing ankle movement and afferent feedback by repetitive electrical stimulation (rENS) of the peripheral nerve to either the agonist plantarflexor muscles (SOL, tibial nerve (TN)) or the dorsiflexor muscle (TA; common peroneal nerve (CPN)). During the breaks between FT sets Group 10 received 1 Hz rENS of TN at 115% rMT, Group 11 received 1 Hz rENS of CPN at 115% rMT and Group 12 received 1 Hz rENS of TN at 90% rMT.

**Figure 6:**
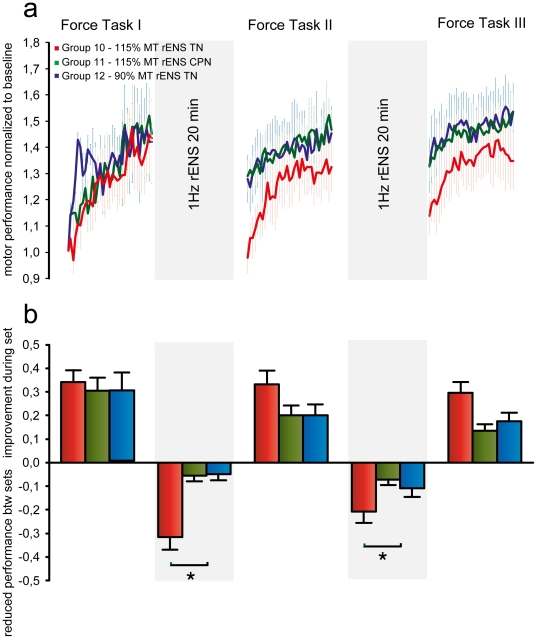
Repetitive electrical stimulation of the nerve to the trained muscles, but not its antagonist causes interference. (**a**) Learning curves for the FT task and effects of rENS during breaks. Performance was normalized to baseline. Curves represent group average FT motor performance Error bars represent s.e.m. During the breaks Group 10 (red) received 1 Hz 115% MT rENS of the agonist peripheral nerve (TN), Group 11 (green) received 1 Hz 115% MT rENS of the antagonist peripheral nerve (CPN) and Group 12 (blue) received 1 Hz 90% rENS of the gonist nerve. (**b**) Increase in FT motor performance during practice and decrease in FT performance during breaks. Bars represent group average ± s.e.m. An asterisk denotes significant difference (p<0.05) in Bonferroni corrected tests.

Figure 6 shows that rENS applied to the tibial nerve at 115% rMT in Group 10 interfered with retention of the ballistic learning, whereas subthreshold stimulation in Group 12 and suprathreshold stimulation of the antagonist nerve in Group 11 did not. A two-way ANOVA on differences in individual FT motor performance within and between sets revealed a significant main effect of SET (F_2,71_ = 86.84, p<0.001) and a significant GROUP×SET interaction (F_4,63_ = 6.548, p<0.001).

All groups improved motor performance with no between-group differences during FT1 (Fig. 6A and 6B). During the first break, FT performance decreased 32±9% in Group 10 to 103% of baseline. This decrease was significantly larger than what was observed for Group 11 (t = 4.586, p = 0.009) and Group 12 (t = 4.667, p<0.007) in which FT performance decreased by 5.5±4% and 5±5% respectively. Between FT2 and FT3 similar observations were made. In Group 10, FT performance decreased 21±6% which was significantly more than Group 11 (7±3%) (t = 2.63, p = 0.028) and Group 12 (12±6%)(t = 2.45, p = 0.042). There were no significant differences between Group 11 and 12.

Analysis of the individual learning curves revealed a significant main effect of both GROUP (F_2,71_ = 3.72, p<0.01) and SET (F_2,,71_ = 29.94, P<0.001) and a GROUP×SET interaction (F_4,,63_ = 3.22, p<0.01). There were no differences between groups in the first ballistic training set. With subsequent practice however, *y0* increased from FT1 to FT2 (t = 2.46, p = 0.017 and t = 2.39, p = 0.013) and from FT1 to FT3 (t = 3.14, p = 0.003 and t = 3.24, p<0.001) for Group 11 and 12 who received suprathreshold CPN stimulation and subthreshold TN stimulation. In contrast, there were no differences in *y0* between training sets for Group 10 who received rENS of TN at 115% rMT, but significant differences from Group 10 to Group 11 (t = 2.70, p = 0.008) and 12 (t = 2.64, p = 0.01) for FT2. Although less pronounced, this was also the case for FT3 *y0* (t = 2.23, p = 0.024) and (t = 2.17, p = 0.03). The parameter estimate *a* displayed a significant main effect of SET (F_2,71_ = 4.05, p<0.01). For Group 11 and 12 the estimate of *a* tended to decrease in FT2 and FT3, but there were no significant differences between groups. In conclusion, retention of ballistic learning was subject to interference selectively by repetitive suprathreshold stimulation of the TN to the FT agonist muscles, whereas this interference was not observed if stimulation was below movement threshold. Suprathreshold stimulation of the CPN to the dorsiflexor muscle TA also did not cause interference.

## Discussion

Practice of a new motor task is usually associated with an improvement in performance. This is generally thought to be mediated by experience-driven changes within the neural circuits involved in the trained task. Indeed, if we stop practicing and return the next day to the same task, we find that our performance has been maintained and may even be better than it was at the first day. This retention is a measure of our ability to form, store and retrieve a motor memory of the task [3], [18]. However, if a second motor task is practiced after initial motor learning interference can occur and consequently motor performance on subsequent occasions may be no better than baseline on day one [2], [6]. How, why and when interference occurs does however remain controversial.

In the present experiments, subjects learned to increase their skill in performing a primary ballistic force task. The “motor memory” of this skill was initially labile and between-task interference occurred in Experiment I if the subjects learned an accuracy-tracking task involving the same movement direction and agonist muscles shortly afterwards.

However, in Experiment II if the competing task was introduced several hours after learning the ballistic task, then no interference occurred. Consolidation may be defined as a set of processes whereby a motor memory is stabilized with continued passage of time and becomes less susceptible to disruption from a competing memory [19], [20]. This implies that the ballistic motor learning consolidated. We speculated that this process of consolidation involves long-term stabilization of synaptic changes that are induced in specific circuits during the period of initial learning. For interference to occur a second task must involve activation of, and learning in, the same neural circuits. The results of Experiment I, II and III were consistent with this.

In Experiment I, the results showed that subsequent practice of a different task, emphasizing accuracy rather than force, caused interference to the extent that ballistic task performance returned to baseline. Importantly, the observed interference was very specific. Interference was only observed when the two tasks involved the same direction of movement thereby engaging the same agonist muscles. When the two tasks were learned using an opposite direction of movement (involving the antagonist muscles) no interference was observed. This demonstrates that interference did not relate to the competing task per se since both groups practiced identical tasks. More importantly, it also demonstrates that interference is specific to the neural circuits encoding a specific movement (direction) and involving a specific set of muscles. Since both Group 1 and 2 were exposed to a potentially interfering intervention comparison between these groups only allowed conclusions on relative interference. However, comparison of these groups to a control group revealed selective interference in Group 1 and no interference in Group 2.

It could be speculated that part of the FT performance gain during practice could be explained by a warm-up effect, which would not relate to learning as such. However, this is not very likely to explain all of the performance gain since significant retention of performance could be observed both 4 hours and 2 weeks later. In addition, warm-up effects would not explain the observed differences between intervention groups.

It is noteworthy that not only did the accuracy task interfere with ballistic learning for Group 1, the ballistic task also interfered with retention of learning in the accuracy task. This effect was also observed specifically for Group 1 in which the two tasks were practiced with the same muscles and direction of movement. Although interference may seem more pronounced in the ballistic task, it is not possible to quantify asymmetry of interference between these tasks. In addition, the ballistic task was practiced for 8 minutes while the accuracy task was practiced for 20 minutes and may also influence the susceptibility to interference. Although the interference effects eliminated improvements due to previous practice it did not affect the ability of the subjects to increase motor performance during the following practice sessions. Rather the interventions may have interfered with the early labile motor memory processes thereby preventing consolidation as indicated by the findings of Experiment II.

The question then becomes: what is the mechanism(s) by which one task interferes with a competing or conflicting task? Naturally multiple mechanisms may contribute differentially, but based on the findings of Experiment I and III interference requires competing learning processes in appropriate motor circuits. In Experiment III the subjects performed voluntary plantar flexions during breaks. These voluntary contractions engaged the same muscles and movement direction as the accuracy task but did not cause interference. So why did the accuracy task lead to interference while the voluntary task did not? During the voluntary contractions subjects did not engage in learning. No feedback was provided, nor were any task constraints reinforced meaning that there was no error signal. The results of Experiment III showed that the observed interference is not related to muscle activation per se, but that interference requires the subject to engage in acquisition of a skill, in order to promote learning in appropriate motor circuits. The lack of interference in the simple voluntary nonlearning task may be due to the fact that this was a task with which subjects were already familiar, perhaps due to the similarity of this task to the ballistic force task.

It has previously been demonstrated that after initial motor learning, competing motor learning [2], [4], [6], pharmacological interventions [12] and rTMS protocols [5] can cause interference. It is important to note that different interfering agents may utilize different mechanisms. One possible mechanism of (retrograde) interference in motor learning may be disruption of motor memory consolidation processes. If interference occurs through disruption of consolidation this would require that there is a limited time window during which the learning of skill B impairs future performance of A in the classical ABA paradigm. There is general agreement that practice of skill B can interfere with future performance of skill A, but there is however a large controversy as to whether this interference actually exhibits a temporal gradient. This may depend on the type of learning.

In Experiment II we demonstrated that learning of the ballistic task did consolidate with passage of time and with increased initial training. Four hours after initial ballistic practice the accuracy task no longer caused interference. This is consistent with the findings of Muellbacher et al. [5] for ballistic motor learning. Although a critical role of a time window for consolidation has been observed previously for ballistic learning as in the present study [5], it has also been a topic of great controversy. In other forms of learning, recent studies have failed to confirm the consolidation window hypothesis [7], [8]. A large part of the studies on interference have focused on visuomotor adaptation, force-field learning and rotation adaptation learning. For these types of learning, interference from competing tasks is persistent and resistance to interference is not observed with passage of time as it is observed as it is in the current study. Miall et al. [21] argued that this persistent interference could be mediated by anterograde mechanisms. In recent studies it has been demonstrated that interference in visuomotor rotation adaptation may be both retrograde, anterograde and due to contextual blocking of retrieval [9]. In the current study, it does not appear that the interference observed in Group 1 was anterograde interference caused by after-effects from accuracy task learning. In that case we should still have observed interference in Group 3 since the time interval from B to A in the ABA paradigm was identical in the two groups. Concerning visuomotor rotation this type of learning does however consolidate over time and with increased initial practice [6].

Different tasks naturally engage different networks and different types of learning may also consolidate differently and have different susceptibilities to interference. This is underlined by a recent study by Baraduc et al. [13], which demonstrated that rTMS of the primary motor cortex disrupted retention of ballistic motor learning but not force-field adaptation learning. This likely relates to differences in underlying networks and the role of the primary motor cortex in the specific type of learning [13]. In the present study, practice of the FT also produced specific increases in corticospinal excitability as evidenced by increased MEP amplitudes for the soleus muscle, but not for the antagonist TA muscle. This would appear significant since soleus was the agonist in the ballistic training task. This finding of increased corticospinal excitability is in agreement with previous studies on motor learning. Numerous studies have previously indicated a role of the primary motor cortex, M1 in skill acquisition [22], [23], [24] - for review see [25] - and recently a few studies have also indicated a role of M1 in early motor memory consolidation [5], [12], at least in certain types of tasks (see [13] for details).

Experiments IV and V tested the importance of motor output and sensory input in causing interference by disrupting the ballistic motor memory. Several studies have documented that low frequency rTMS can reduce cortical excitability transiently [5], [14], [15], [16], [26] for review see [17]. Like Muellbacher et al. [5] we found that application of suprathreshold 1 Hz rTMS of the contralateral motor cortex caused interference. Consistent with the findings of the present study Muellbacher et al. [5] found that 4 hours after initial learning of the ballistic task, rTMS did not cause interference. In control experiment rTMS of the occipital and dorsolateral prefrontal cortex did not cause interference and this led to the interpretation that M1 is involved in the early establishment of memory of the ballistic motor task following training. However, we also found that subthreshold TMS, which is also known to activate M1 and corticospinal outputs [17], [27], [28], failed to interfere with motor memory. This difference between rTMS effects could simply be because subthreshold rTMS failed to activate sufficient neurones to produce interference, or it could indicate an important role for re-afferent feedback from the movements evoked by suprathreshold stimulation.

Consistent with the TMS results presented here (see fig. 5) Lang et al. [16] demonstrated that corticospinal excitability was suppressed more after suprathreshold as compared to subthreshold rTMS, but in addition, the evoked motor potentials were also suppressed following suprathreshold 1 Hz electrical stimulation of the peripheral nerve (rENS). The interpretation of these findings was that the intensity of stimulation has an impact on the after effects of rTMS and that reafferent feedback may contribute to the stronger suppression of corticospinal excitability observed following suprathreshold 1 Hz rTMS compared to subthreshold 1 Hz rTMS.

In fact, Experiment V suggested that the (re)afferent feedback could be highly important since ankle plantar flexion produced by peripheral nerve stimulation of the agonist nerve (rENS), which generates strong sensory input, also interfered with motor memory consolidation. Again the interference effect observed following rENS was very selective and did not occur when the antagonist nerve was stimulated or when subthreshold intensities were used.

Why then did a non-targeted voluntary movement of the ankle, which produces sensory input similar to that evoked by peripheral nerve stimulation at 1 Hz, fail to interfere with the formation of motor memory? The difference may be that sensory feedback produced by volitional movement is predictable whereas that produced by peripheral nerve stimulation alone (as well as input produced by suprathreshold TMS) is unexpected. In Experiment III the sensory feedback was generated naturally by voluntary movement and so would not conflict with the expectations. Peripheral nerve stimulation leads to artificially generated sensory signals. In the context of learning, the CNS may interpret this as an error signal indicating a discrepancy between expected movement and the actual movement signalled by sensory feedback. Recent research has suggested that the cerebellum has a crucial role in detecting such discrepancies, assists the cerebral cortex in transforming sensory signals from spinal modules to motor-oriented commands and that it updates motor programmes so that future movements are performed more optimally [20], [29]. In line with this notion, Chen et al. [30] recently demonstrated that disruption of the human cerebellar thalamus which relays cerebellar signals to motor cortex significantly impaired the ability of the brain to form internal models of action.

Several studies have suggested that motor skill acquisition progresses in multiple dissociable stages, which have different sensitivity to feedback error signals. Smith et al. [31] proposed that at least two distinct processes with different learning rates and different capacities for retention are involved in motor learning. This may also relate to susceptibility to interference. One process proceeds rapidly but has poor retention. This phase is hypothesized to depend strongly on feedback error signals and may be located in the cerebellum. The other process evolves slowly and responds weakly to error but retains information well. Hadipour-Niktarash et al. [32] recently suggested that M1 contributes to the slow processes that maintain motor memory. Furthermore recent experimental studies highlight the key role of the cerebellum in modulating excitability of M1 after sustained peripheral stimulation as in the present study [29].

We consequently propose that unexpected sensory input can be interpreted as an error signal to update the synaptic efficiency in the neuronal circuitries in the sensory-motor system subserving motor performance during and following practice. This disrupts any ongoing plastic changes from previous learning unless these have been consolidated by changes in protein synthesis. Indeed, the remarkable muscle specificity that we have observed at least at the ankle joint suggests that the some of the synaptic changes could occur at lower levels of the motor output such as M1 or the spinal cord.

The present findings add knowledge to the literature on interference and consolidation in motor learning in several respects. The results demonstrate that the observed interference effects are remarkably specific, consistent with the idea that interference occurs in neural circuits that are involved in a specific movement and activation of individual muscle synergies. Two important results reinforce each other: Experiment I and III demonstrated that between-task interference requires identical or overlapping circuits to be engaged in competing motor learning processes; Interference does not occur when learning is not involved. In such cases, there is no error signal and therefore no competing motor memory consolidation process. Secondly, the present study is, to our knowledge, the first to demonstrate that peripheral nerve stimulation may cause interference possibly through disruption of early motor memory formation. This emphasizes the importance of sensory feedback error signals in interference and motor learning.

## Methods

### Participants

Sixty-one adults aged 20–42 years, (25±4, mean + s.d.), 39 males and 22 females trained a ballistic force task (FT) involving rapid plantar flexion with the left ankle joint. Participants were untrained to moderately trained and had no known medical or neurological conditions that could impair motor learning or performance. Participants who prior to participation had a history of training ballistic plantar flexion movements were excluded from the study. Participants were randomized into twelve different training groups described in details below (See fig. 1C). Participants in Groups 3, 4 and 5 only participated in one experimental session. Participants in all other groups were included in two different training groups, so that N = 8 for all groups except Group 1 and 2 in which N = 9. A minimum of 2 weeks between each participant's experimental sessions was given to minimize the influence of the first test on the second test. Baseline performance in session 2 was however significantly better than in session 1. This demonstrates long-term retention of the ballistic FT learning. The motor performance measures reported in the results section were always normalized to the baseline performance in the individual test and although the reuse of subjects could potentially affect the amount of interference observed in individual subjects, the reuse of subjects does not affect the conclusions of the study, since subject allocation was randomized and marked differences were observed between different intervention groups regardless of prior experience with the task. Written informed consent was obtained from all participants before the experiment. The experiments (KF 01-131/03) were approved by the local ethics committee of the Capital Region of Denmark (De Videnskabsetiske Komiteer for Region Hovedstaden) and followed the regulations expressed in the Helsinki declaration (1964).

### Force Task

All twelve groups performed and practiced a ballistic force task (FT) consisting of 2 or 3 sessions of 35 ballistic isometric plantar flexions of the ankle joint. Group 5 performed 45 ballistic plantar flexions in the initial training set. Before the practice started participants were instructed to perform a 5 min. warm-up session on a bicycle ergometer. During motor practice the participants were seated in a custom build chair with their left foot attached to a force pedal. Before training subjects were instructed how to perform the task and allowed two test contractions in order to become accustomed to the task. Participants were instructed to produce as much torque as possible by pressing the force pedal (isometric contractions) using plantar flexor muscles within 250 ms, then relax and return to baseline within a total time window of 500 ms. Isometric conditions where chosen in order to minimize any contribution of antagonist muscle activation such as that seen as part of a triphasic activation pattern during rapid concentric movements in the upper limb [33].

The participants performed one ballistic isometric plantar flexion every 10 s. The participants were given visual feedback on a monitor placed in front of them. The monitor displayed a window with a trace of the force applied during each contraction, the specified time window for contraction and a continuously updated trend plot of the FT performance in all trials obtained during the whole set. The participants were instructed in each trial to perform a maximal ballistic contraction and to increase maximal force across trials. Trials in which a countermovement (defined as a downward deflection of the baseline) or “false start” (defined as an upward deflection of the baseline preceding the actual contraction) occurred were not included in the analysis. If such trials were noted during the experiments an extra trial was performed. During all training sessions participants were verbally encouraged to improve their performance as much as possible. The three FT training sets (FT1, FT2 and FT3) sessions were separated by periods of 20 minutes. During these periods the training groups were subjected to different interventions.

### Accuracy task

During the two 20 min breaks between FT training Group 1 and 2 trained a second motor task. Contrary to the force task this second task emphasized maximal accuracy. The accuracy task (AT) training was performed in order to evaluate the effect of learning a second motor task on the recent motor learning from the first task. Secondly, it was the purpose to investigate whether any observed disruptive effects on motor memory consolidation were general or muscle/movement specific. The AT involved visuomotor tracking of a computer generated sinusoid curve with one cycle pr 10 sec on a monitor using only the plantar flexion (Group 1) or dorsiflexion (Group 2) force signal. The curve was displayed in windows of 8 seconds each and each subject performed a total of 120 windows in each 20 min period. Before beginning of the training subjects were instructed how to perform the AT and allowed two test trials in order to become accustomed to the task. The subjects were instructed to keep the force signal as close to the target curve as possible at all times and were verbally encouraged to improve their performance as much as possible. The maximal force produced during the AT task was very low (5% of MVC).

### Passage of time between initial learning and learning of a competing task and effect of extra practice

In Experiment II (Group 3 to 5) we investigated whether the FT learning consolidates over time and with increased initial learning. To elucidate whether the susceptibility to interference by learning a competing task was affected by passage of time Group 3 had a break of 3 hours and 40 minutes after training the FT. Following this break subjects engaged in the AT training involving the same movement direction and agonist muscles (i.e. plantar flexion). Immediately after this the subjects engaged in a second FT training set. Group 4 did not train the AT. Instead, these subjects had a break of 4 hours in-between FT training sets. In Group 5, subjects also had 4 hours break in-between FT sets, but the amount of training in the initial FT set was increased from 35 trials to 45 trials in order to investigate whether FT learning consolidated with increased initial training i.e. if extra learning affected retention.

### Voluntary contractions of agonist muscles during breaks

In order to investigate whether any effects observed in Group 1 or 2 could be related to fatigue or simple use of the agonist rather than learning a different task, Group 6 performed small isometric contractions (<5% of MVC) of the soleus muscle at a frequency of 1 Hz during the 20 min breaks in between force task training with no learning requirement and no feedback on performance.

### Repetitive transcranial magnetic stimulation and peripheral nerve stimulation during breaks

To further elucidate the mechanisms and susceptibility to interference and the role of motor output and sensory feedback different interventions were applied during the breaks in between FT sets. Group 7 and 8 received 1 Hz rTMS of M1 for 20 minutes at intensities of 115% and 90% rMT for the SOL muscle. Group 9 received sham TMS. Group 10 and 11 received rENS of the tibial nerve at an intensity of 115% and 90% SOL rMT. Group 12 received 1 Hz rENS of the common peroneal nerve at an intensity of 115% TA rMT. These different stimuli were applied to evaluate the effect on newly formed motor memory. Each participant received a total of 1200 stimulations at a rate of 1 Hz during each pause. The stimulus parameters for the peripheral nerve stimulation were chosen in order to evoke motor responses and (re)afferent activation corresponding to the rTMS evoked responses.

### Recording and stimulation procedures

Electromyographic (EMG) recordings from the TA and SOL muscles were obtained with bipolar surface EMG electrodes (0.5 cm diameter of electrodes; 2 cm distance between electrodes; Blue Sensor, Ambu Inc.,USA) over the belly of the muscles. Torque was measured with a custom build force pedal with a build in strain gauge. The EMG signals were amplified (×2000), using custom build EMG-amplifiers, filtered (band-pass, 25 Hz to 1 kHz) sampled at 2 kHz, and stored on a PC for off-line analysis (CED 1401+ with Signal 3.09 software, Cambridge Electronic Design Ltd., UK).

Magnetic stimuli were delivered to the contralateral (right) hemisphere primary motor cortex (M1) by a Magstim Rapid^2^ stimulator (Magstim Company Ltd., Whitland, UK) via a custom-made 90 mm figure-of-eight coil (batwing design, Magstim Company Ltd., Whitland, UK). The optimal position of the coil for eliciting motor evoked potentials (MEPs) in the SOL muscle was established through a mini-mapping procedure of M1 and the coil was placed on the scalp over the hot-spot of the SOL representation with the handle of the coil pointing horisontally backward, so that the current in the brain flew in posterior-anterior direction. Motor threshold (MT) was defined, as the minimum intensity required to produce MEP amplitudes larger than 50 µV in 3 out of 5 trials.

During all experiments involving TMS a Brainsight^tm^ image guided TMS navigational system (Brainsight-Frameless 1.7.7 Rouge Research, Canada) was used to monitor the position of the coil. rTMS experiments were performed in accordance with current safety recommendations [34]. In the sham TMS condition (Group 9) a magnetic coil was placed on the scalp of the subject and a second coil was placed above this coil. Stimulations were delivered only through the second coil, thus not activating the motor cortex. TMS recruitment curves were obtained through application of stimulation intensities from 0.8–1.4 MT in steps of 0.1. Stimulations were delivered in a random sequence with 4 s inter stimulus interval. 5 stimuli were obtained at each intensity and the MEP was measured as the average peak-to-peak amplitude of five trials.

In Group 10 and 11 soleus Hoffmann reflexes (H-reflexes) were elicited by electrical stimulation of the posterior tibial nerve (PTN) with a 1 ms square-wave pulse (model DS7A Digitimer, US) in the range 10–50 mA using a custom build ball-shaped mono-polar electrode placed in the popliteal fossa. The anode was placed proximal to the patella. H-reflex threshold was defined as the minimum intensity required to evoke a H-reflex visible in the online soleus EMG. In Group 12 activation of TA was elicited through electrical stimulation of the common peroneal nerve (CPN) with a 1 ms square-wave pulse (model DS7A Digitimer, US) in the range 10–50 mA using bipolar electrodes (Blue Sensor, Ambu Inc. USA) The anode was placed distal and lateral to the insertion of the patellae ligament and the cathode just below the neck of the fibula above CPN. In TA it was not possible to evoke a clear H-reflex in any participants. Consequently stimulations were applied at 115% M-wave threshold defined as the minimum intensity required to evoke an M-wave in the online TA EMG (for methods see e.g. [35]).

### Data analysis

Motor performance in the force task (FT) was calculated as the peak ankle torque within the time window. The torque produced in each trial throughout training was normalized to baseline performance i.e. the torque produced in the first trial during training. Motor performance in the AT task was calculated as the mean difference between the position of the target sinusoidal curve and the force signal in each trial. This average deviation from optimal performance (error) was also normalized to baseline performance. This normalization of motor performance to baseline was performed in order to allow comparison. For quantification of improvement of performance during training sets and loss of performance between sets an average of the first 3 trials and the last 3 trials in each set was calculated respectively. Statistical analysis was performed on the data using Sigmaplot 11 (Systat Software Inc. USA). Before statistical comparison, all data sets were tested for normal distribution by a Kolmogorov-Smirnov test. Motor performance in the force task and the accuracy task in the different groups were compared by two-way analysis of variance (ANOVA) with time (set) and groups as factors. Initially, the increase in motor performance during FTI was tested against baseline for all groups. Following this, separate tests were completed for each of Experiment I to V. In these tests a two-way ANOVA was used to test for differences in force task performance within and between groups during FTI, FTII, FTIII and during the break between sets (FTI-FTII and FTII-FTIII). Post hoc pairwise comparisons were performed with Bonferroni tests. In Experiment I the statistical FT analysis included data from Group 1,2 and 9 in order to enable comparison of interference effects to a control (sham) group. It should also be noted that Experiment II consisted of a different number of sets and hence also a different number of statistical comparisons. A corresponding two way ANOVA was set up for the AT performance in Experiment I, while improvement in AT performance in Experiment II was tested as a paired t-test.

To further elucidate how the different interventions caused interference with motor learning all FT learning curves were fitted to a three-parameter power function y = y0+ax^b^. Again, a two-way ANOVA was performed for each experiment on the parameter estimates with group and set as factors. MEP amplitudes were normalized to MEP_maxpre_ to allow comparison. MEP amplitudes were compared by a two-way ANOVA with treatment and time as factors. In all statistical tests multiple comparison analyses, post hoc Bonferroni tests were performed for all pairwise comparisons. All values are reported as mean ± s.e.m. unless stated otherwise. In all tests, statistical significance was assumed if p<0.05.
